# Exploring healthcare workers’ perspectives of video feedback for training in the use of powered air purifying respirators (PAPR) at the onset of the COVID-19 pandemic

**DOI:** 10.1186/s12909-022-03742-8

**Published:** 2022-09-23

**Authors:** Mary Wyer, Ruth Barratt, Su-yin Hor, Patricia E. Ferguson, Gwendolyn L. Gilbert

**Affiliations:** 1 Sydney Institute for Infectious Diseases, The University of Sydney, Level 6 Block K Westmead Hospital, Westmead, NSW 2145 Australia; 2 New South Wales Biocontainment Centre, Westmead Hospital, Cnr Darcy and Hawkesbury Rds, Westmead, Australia; 3 Westmead Clinical School, Faculty of Medicine and Health, The University of Sydney, Sydney, NSW Australia; 4 Centre for Health Services Management, Faculty of Health, University of Technology Sydney, Sydney, NSW Australia; 5 Department of Infectious Diseases, Westmead hospital, Cnr Darcy and Hawkesbury Rds, Westmead, Australia

**Keywords:** Video Feedback, Powered Air Purifying Respirator, Personal Protective Equipment, Education, Medical, Qualitative Research, Interviews, Learning

## Abstract

**Background:**

With the advent of COVID-19, many healthcare workers (HWs) in Australia requested access to powered air purifying respirators (PAPR) for improved respiratory protection, comfort and visibility. The urgency of the response at our hospital required rapid deployment of innovative training to ensure the safe use of PAPRs, in particular, a video-feedback training option to prepare HWs for PAPR competency.

**Aim:**

To explore the feasibility, acceptability, and utility of video-feedback in PAPR training and competency assessment.

**Methods:**

Semi-structured interviews were conducted with 12 HWs, including clinicians from Intensive Care, Anaesthetics and Respiratory Medicine, at a large teaching hospital in Australia.

**Findings:**

Participants believed that the use of video-feedback in PAPR training was feasible, acceptable and useful. They described a variety of benefits to learning and retention, from a variety of ways in which they engaged with the personal video-feedback. Participants also described the impact of reviewing personalised practice footage, compared to generic footage of an ideal performance.

**Conclusion:**

By conceptualising video-feedback using a pedagogical approach, this study contributes to knowledge around optimising methods for training HWs in PPE use, particularly when introducing a new and complex PPE device during an infectious disease outbreak.

## Introduction

The SARS-CoV-2 pandemic has presented many challenges for infection prevention and control (IPC), including changes to routine protocols for the use of personal protective equipment (PPE). The rapidly emerging pandemic, and uncertainty about disease risks, contributed to healthcare workers’ (HWs) anxiety about their own health and safety when caring for COVID-19 patients [1]. Confidence and competence in using PPE are key factors in allaying those fears. However, although PPE training is recognised as an essential IPC measure [2, 3], many HWs do not receive regular PPE updates or are unfamiliar with novel equipment used during outbreaks [4]. Powered air purifying respirators (PAPR) have been adopted, increasingly, in Australia, during the pandemic and the urgency of the situation required rapid deployment of innovative HW training methods, to ensure their safe and effective use.

HWs conventionally use P2/N95 particulate respirators as protection against airborne infections, such as pulmonary tuberculosis, measles or chickenpox and for aerosol-generating procedures in patients with other respiratory diseases [5]. During the pandemic, prolonged use of P2/N95 respirators, which requires a tight seal on the face, has caused significant discomfort and facial skin damage [6–8]. A PAPR is an alternative respirator, previously reserved for managing high consequence infectious diseases (HCID), such as Ebola virus disease. With the advent of COVID-19, many HWs, including those at our hospital, requested access to PAPR for greater protection, comfort and visibility, especially in settings such as intensive care units, where prolonged respirator use is required [9–11].

Unlike disposable P2/N95 respirators, PAPRs comprise multiple components that must be assembled, donned correctly, and removed safely, in correct order. This adds complexity and exposes HWs to increased risk of self-contamination and occupationally-acquired infection [12, 13]. Optimal PAPR training should therefore incorporate donning/doffing practice, using checklists, with a trained observer [14]. This level and frequency of PPE training demands significant human and equipment resources and time, which is challenging during a pandemic.

During the first half of 2020, the Biopreparedness team at a designated HCID referral hospital in Australia offered a video-feedback option in the PAPR training program, for HWs from ICU, anaesthesiology, and respiratory medicine. HWs were required to pass a competency assessment before using PAPRs independently. To prepare for the assessment, they were sent a generic, commercial video to watch before face-to-face practical training. During the practical session, HWs were offered the opportunity to have their practice videoed by the trainer. The video footage (a 10–15 min clip) was subsequently shared with them privately to view in their own time, as a source of feedback. This option was intended to augment their learning and preparation for PAPR competency assessment, without the need for repeated face-to-face training sessions, and as a reference for future use.

Video-feedback is a common training tool in healthcare, that can develop and improve HWs’ communication and interaction skills [15–17] and assessment practices [18, 19]. A key aspect of video-feedback is the provision of footage of participants’ *own* practice, which enhances learning and self-knowledge through the personal salience of the feedback [15, 20]. In 2010, a metanalysis of video-feedback [15] found that, when using pedagogically sound approaches, that incorporate the psychological safety of participants, video-feedback can have positive effects on learning. Further studies to clarify which approaches are most effective were advocated. Since then, studies have demonstrated that using video-feedback in debriefs following simulation, role play, OSCE (Objective Structured Clinical Exams) assessments and real time practices can improve students’ performance, self-efficacy and ability to act on feedback [17, 21, 22].

The aim of this qualitative study was to explore HWs’ perspectives on the feasibility, acceptability and utility of video-feedback in PAPR training and competency assessment, early in the COVID-19 pandemic. The research objectives focused on the practical barriers and facilitators when using a digital platform to share personalised videos and our findings augment the growing literature that supports the use of video-feedback in training.

## Methods

### Study design and setting

Semi-structured interviews were conducted with participants who worked at a large teaching hospital in Sydney, Australia. Selected staff who received training and competency assessment in use of CleanSpace® HALO™ PAPR, between March and June 2020, were invited to participate in the study. The research team’s research and clinical experience included IPC, infectious diseases, social science and video-reflexive methodologies. One researcher was a member of the Biopreparedness team involved in the initial training sessions.

### Recruitment, participants, and consent

Eleven nurses and 14 doctors, who had undertaken this training for the first time, were invited to participate in the study, by a member of the Biopreparedness team not involved in training. Those who expressed interest were contacted by a researcher, who explained the study, answered any questions and provided participant information and consent forms for review (hard copy or via email).

Participants were given at least two weeks after initial discussion with the researcher, to consider participation. Researchers then obtained written consent, personally, at the participant’s place of work and arranged an interview time and place. Participants could withdraw from the study at any time, and their data could be deleted if requested. Twelve of the 25 HWs contacted for recruitment consented to be interviewed, including five ICU doctors, four ICU nurses, two anaesthetists and one respiratory consultant.

### Data collection

Semi-structured interviews were conducted between November 2020 and May 2021, in person at the hospital or via phone or web-conference (using Zoom, version 5.7.6). An interview guide based on the research aims was used to guide the discussion. Interviews of 15–30 min were audio-recorded, then transcribed verbatim using a confidential transcription service. Transcripts were deidentified and a participant code assigned.

### Data analysis

Interview transcripts were analysed independently by two researchers, using NVivo software (QSR International Pty Ltd. Version 12.6.0) to organise and code the data. Transcripts were read several times, and coded using the research aim as a guide [23]. Further thematic analysis was then conducted to identify patterns beyond the initial research aims.

## Findings

Overall, participants felt that the use of video-feedback in PAPR training was feasible, acceptable and useful. They described various ways it could be used for ongoing learning, and the positive impact of personalised footage on their practice, compared to generic footage of procedures. A key theme was the *affordance* of video-feedback; i.e. the relationship between the properties of an object (the video clip), and the capabilities of the person (HW), that determines how the object can be used. [24]

Below, we describe the themes and sub-themes that emerged (Table 1), with illustrative quotes from participants.

**Table 1:** Themes and subthemes

Theme	Affordances of video-feedback	Effects of personalised footage	Feasibility and acceptability of the video-feedback process
Sub-themes	•Richer representation of a complex practice •On-demand access to footage	•Viewing footage of self vs other •Practice vs perfect	•Consent and confidentiality •Accessing and viewing video links •Feasibility of video-feedback for educators

### Affordances of video-feedback for learning

#### Richer representation of a complex practice

Many participants reported that re-watching the PAPR donning and doffing processes on video was more engaging than reviewing a written protocol. It was not only a faster way of revising but also the audio-visual format represented complex elements better than they could be explained in writing.

*It is much more helpful than the word description* [competency checklist]. *Just to go through what you actually did* [*...*]. *I’m pretty time poor* [*...*] *and I really don’t have time to sit down and go over all these things* [*...*] *and being able to sit there and watch the video, I thought was* [*...*] *much better than me sitting down and reading it. (#2, Doctor)*

*It’s not all that straightforward, like, you know, just don PPE. Because you have this equipment which needs to go on and then other layers of PPE on top and underneath etc. (#5, Doctor)*

Participants felt the format was suitable for ‘visual learners’, and easier to remember, allowing the process to “*stick in my memory a bit more” (#3, Nurse)*. A similar point was made about verbal prompts from the trainer, captured on the footage.

*The video focused on me undergoing training and the trainer being around and explaining the steps* [*.*..] *voicing and prompting in the background, which also helped* [*.*..]. *Little prompts here and there about whether the fit was adequate or not and things like that. (#5, Doctor)*

This sub-theme describes how the video, as a modality, represented the complex donning and doffing process, as well as the training experience, in greater detail for participants.

#### On-demand access to footage

The ability to view the footage on demand was also beneficial. For instance, several participants watched (or re-watched) the footage to prepare for their competency assessments, or when they needed to use the PAPR in practice. This helped them quickly recall the process and reminded them of parts they had found difficult during training.

*So, I watched it immediately when I got the link* [*.*..] *and then actually I went back to it in a few days to watch it again. And then after, I almost forgot about it, but then* [when] *we had to use* [it], *I went back to it, and I watched it again (#9, Doctor)*

The ability to review the footage was particularly helpful for some, who felt they had forgotten much of the information given during training, because of the quantity of new information shared during the pandemic, and the passing of time.

*I couldn’t actually remember half of the stuff I was being told, because there was a lot of information to absorb…and particularly during COVID, there was just so much new information coming all at the same time. But* [*.*..] *the most useful part I found was that I was able to access* [the video] *the night before and I could watch it. (#2, Doctor)*

Another benefit of on-demand access was that participants could replay their footage as frequently as needed, and pause to cement or to refresh their understanding of the procedure.

*You can stop* [the video]*.* […] *you can think. And then go back. If you look at the video multiple times, you are going to remember the steps when you are doing it by yourself. (#1, Doctor)*

*I could look at the video and then go through the steps without having anyone else there, so to speak. So instead of having to come to work, find someone who is trained in training, I could watch the video and go, oh, that’s right, that’s the second step not the third step. (#6, Doctor)*

One caveat noted, however, was that the continued use of the footage depended on protocols remaining the same.

*Well, if we have to go and start using* [PAPRs], *then yes, I believe I will* [review the video] [*.*..] *as long as the actual procedure doesn’t change much (#6, Doctor)*

This sub-theme highlights that HWs valued the video as a learning resource, as they could review it when, and as often as they wished, to cement or refresh their learning.

### Effects of personalised footage

#### Viewing footage of self vs other

Most participants agreed that being videoed and watching footage of *themselves* donning and doffing was important, rather than a generic video of someone else. In the first instance, awareness of being videoed made some participants more attentive to detail *during the training itself*.

*While I was doing it,* [I was] *being filmed…so obviously* [I was] *a bit more conscious about it* [*.*..] *I just wanted to make sure I’m doing it right* [*.*..]. *So, try and remember, trying to be as best I can be. So, I think the video is probably good in that purpose (#3 Nurse)*

Another effect was that participants felt they learned something about their own practice. Participants commented on this, not just for the video-feedback of PAPR training, but also how it might be used to improve other aspects of their work.

*It is hugely insightful, I found, when you see yourself. You do it every day, and you don’t really realise sometimes you might be doing something just that ever so slightly different. Not necessarily wrong, but just different, and it maybe there may be an easier way of doing it perhaps or like a better way. Or sometimes you could be doing it wrong, and you don’t realise it. (#9, Doctor)*

Importantly, the video footage included not only the participant’s donning and doffing practice, but also feedback given, such as verbal prompts, where the educator might point out issues particular to the learner, or actions that other people found difficult. This meant that participants were reviewing and recalling a (sometimes imperfect) *personal experience*, which may have been more salient, and therefore impactful, than a generic ‘perfect’ procedure.

*I was able to recall what happened on that day. Unlike watching someone else just going through the motions* […] *And then it brought back memories, at certain juncture, where I had made a mistake and they could have corrected me there. So that refreshed my memory and I probably did not repeat that mistake again during assessment. (#5 Doctor)*

This sub-theme describes the particular salience, for HWs, of being featured in the footage – in both filming and watching it – as being valuable for learning.

#### Practice vs perfect

Participants reported different opinions about having ‘practice’, rather than ‘perfect’, footage to review. As noted, many participants identified the particular salience of watching themselves make mistakes, or perform actions needing improvement, as valuable for their learning.

*I had trouble…getting a certain clip on something. And I remember* [Educator] *said to me at the time* [*.*..] *“People have difficulty with this bit, they always sort of do it wrong and you’ve just got to do ‘that’”. And when I watched the video – watched the error that I made, that made me take note to remember that* [*.*..]. *It sort of personalises it for you* [*.*..]. *I think* [it is a] *more informative learning tool than watching someone else make a mistake* [*.*..] *there’s nothing like watching yourself making a mistake and remembering that. (#2, Doctor)*

A few participants, including the two who had not watched their own footage, suggested that a generic video demonstrating the correct procedure could have been more useful, particularly as a refresher.

*I do like a generic video* [*.*..]. *They’ve made sure that* [*.*..] *all the steps are correct. And then you can kind of recreate it. While* [in my own footage] *I might be pausing for a second, listening, and then fiddling around the wrong way. (#11, Nurse- did not watch own video)*

For other participants, watching their own practice, even if imperfect, gave them confidence in their ability to perform the procedure safely, both during their competency assessment, and when caring for an infectious patient.

*Watching myself do it, gave me some confidence in the fact that, “Yes I can do it!”. And whilst it might not be perfect, I was still getting it as perfectly as possible and certainly I knew at the end of watching the video that I was not going to be breaching any kind of protection to myself or to the patient. And I knew the points where I had to be careful, that I had to be more mindful. (#9, Doctor)*

This sub-theme describes the different potential benefits for learners, of capturing an imperfect learning experience on video, as compared to an idealised demonstration, for subsequent review.

### Feasibility and acceptability of the video-feedback process

#### Consent and confidentiality

Although our participants did not express concerns about being videoed or the way their personal footage was sent to them, some suggested others might have concerns about the potential for the footage to be distributed more widely.

*So, if you video a person and you send their video to them it’s the best way forward. But I don’t think people will agree, not everyone, to be videoed and then to be sent out widely for education purposes. (#1, Doctor)*

#### Accessing and viewing video links

All participants reported being able to easily access the video link sent by email, although one noted it might be difficult to find in future, given the volume of emails received by staff.

*I’ve got something like 8000* [emails] *stored so, I would need a way of finding it. (#7, Doctor)*

Most participants viewed the footage in the days after receiving it—usually the entire clip. Participants who did not view the clips gave time constraints as their main reason, although some also already felt confident in their practice.

*So, the reason I honestly didn’t watch it is I just didn’t have time* [*.*..]. *With COVID happening, we’re all just trying to train the staff.* [*.*..] *also I was pretty confident in putting it on the first time* [*.*..]. [I thought] *“Do I really need to watch myself do it again?” (#11, Nurse)*

#### Feasibility of video-feedback for educators

Participants recognised the extra time and resources involved in educators’ adopting video-feedback, while also noting its suitability, given the increase in online learning due to the pandemic.

*I guess my concern would be how practical* [videoing individuals] *would be in the day-to-day training of people because, again, it requires a bit of resources to set that up, and I think a better use of resources would be in training a buddy. (#8, Doctor)*

*I think now, because we’ve integrated more online learning – because of COVID – into our way of teaching,* […] *I’d sit there and watch* [videos] *a bit more regularly now. (#11, Nurse)*

Future uses of video-feedback suggested included training in the use of other devices or close-up footage of difficult PAPR actions, like unclipping the mask from the motor unit, or expanding its bellows. Participants also saw value in using video for self-assessment in simulated exercises and formal assessments.

*So, this year, during COVID* […] *we used our SIM Centre* [to] *run OSCEs*. *And you know, it hasn’t occurred to us yet about recording it, but it’s probably something that could be done, and I wonder whether that adds an additional element for people to self-assess their performance. (#2, Doctor)*

*Look, the bottom line is that with regards to the videoing it and playing it back and allowing you to watch it, I think it’s a good idea. I think it’s really useful for PAPR or any other training, that you want to* [do]. *(#5, Doctor)*

The above three sub-themes describe the logistical and ethical considerations in using video-feedback, as well as its potential uses for training in other topics, and in combination with other training methods in health professional education.

## Discussion

Our findings generally support the use of video-feedback in PAPR training as feasible and acceptable. Participants in the study received face-to-face and video-feedback combined, which was described as an engaging and meaningful way to learn – as found in other studies of video-assisted learning in healthcare [25]. Nevertheless, there were caveats, including the need to maintain participant privacy, and time constraints which prevented some staff from reviewing footage.

Heavy workloads and associated time pressures are often described as barriers to continuing education and training in healthcare [26], which have been exacerbated during the pandemic [27, 28]. Conversely, participants also described the use of video-feedback as time-saving, as it allowed flexibility to review procedures in their own time, at their own pace, without needing to consult an educator. These affordances of video footage have been identified previously in the medical education literature [18, 29].

For educators, the time and resources involved in providing VF are not significantly more than those necessary for face-to face training. It requires an initial investment in equipment (camera and tripod), secure data storage and file sharing software but, once established, it takes no extra time to record training. Downloading and sharing footage over the internet requires much less time than repeated training face-to-face sessions [18]. Another benefit of video-feedback is its suitability during a pandemic, when online learning is more common and necessary to avoid workplace transmissions.

Shepherd and Burton [30] argue for the primacy of a conceptual framework when designing and delivering simulated activities in healthcare education. Our video-feedback method was based on video-reflexive ethnography [31] which we have used successfully to improve HWs’ and patients’ understanding and practice of IPC [21, 32–36], including PPE training with medical interns [21]. Video-reflexive ethnography harnesses the learning that occurs as a *response* to being confronted with habituated ways of practice. Video footage of everyday practices is shown to participants and their colleagues, in facilitated discussions, enabling them to grapple with the complexity of their work, and increase their understanding, agency and control over what practices work well, and what needs to change [31].

In this study, the theoretical underpinning is demonstrated in the use of video, to represent the physical intricacies of donning and doffing PAPR, which captures more detail than the written procedural checklist provided to clinicians for this new equipment. Researchers have recognised the limitations of checklists for addressing complex practices and problems (e.g. [37, 38]) including Iedema et al. [31] who promotes video-reflexive feedback instead.

The video-reflexive underpinnings are also reflected in recording participants’ practice sessions, including teaching prompts, mistakes, and other personal details, rather than generic footage of an ‘ideal’ performance. While a few participants expressed a preference for an ideal performance for revision, many acknowledged the value of personalised footage, which made it possible for them to “hear again, to feel again and to think, question and remember again” (MacDougall in [31] p. 27). Iedema et al. [31] describe this as part of the “hologrammatic” (p. 27) effect of video, where, upon reviewing their own footage, participants can recognise not only what they see on the screen, but also what is not explicitly shown: the wider experience of what happened on the training day; the context in which training and future PAPR use will take place; potential personal improvements; and, as some participants noted, their habituated, unquestioned ways of doing things. Once these habits are recognised, they become malleable to change.

Our findings are limited by the small sample size and one training program, in one hospital. Nonetheless, they contribute to the scant research on the acceptability, feasibility, and utility of video-feedback on skill-based learning, that has been primarily conducted using surveys [16]. In-depth interviews provide a more detailed understanding of what aspects of video-feedback HWs find beneficial. More studies are needed to investigate HWs’ satisfaction with different styles of video-feedback, particularly for situations where rapid training is required to roll out new equipment. Further research could also explore the use and impact of collaborative (and peer) video-feedback, which is closely aligned to the *collaborative* principle of video-reflexive methodology [31], and can have positive effects on skill-based learning [16].

## Conclusion and recommendations

Based on our findings, we make the following recommendations for educators who wish to incorporate personalised video-feedback into education and training programs. It was suggested that some people might object to being videoed for training purposes, so consent should be obtained, and an alternative provided, such as a generic video demonstration (e.g., available on a facility education web page). Since healthcare procedures are regularly revised, an advantage of a generic video is that it can be updated and accessed readily, if HWs need to tweak their practice and there is delay in accessing face-to-face refresher training or competency.

Factors that could improve learning, when reviewing the footage, include showing close-ups of fiddly or awkward actions (e.g., unclipping mask or pressing buttons out of eyeline), and retaining auditory teaching prompts in the video footage. The addition of a self-scoring tool that learners can complete when watching their training video has recently been found to result in a measurable and sustained improvement in the safety behaviours around PPE [39]. Educators must also consider how personal footage (in the form of large video files) might be securely transferred to trainees, for example, using an encrypted internet-based computer file transfer service or a via a facility’s internal shared drive.

By conceptualising video-feedback within a pedagogical approach, this study contributes to knowledge around optimising methods for HWs’ PPE training, particularly when introducing a complex new device during an emerging infectious disease outbreak/pandemic, as well as in routine training.

## Data Availability

The datasets generated and/or analysed during the current study are not publicly available as individual privacy could be compromised but are available from the corresponding author on reasonable request.

## Abbreviations

HW: Healthcare workers
HCID: High consequence infectious diseases
IPC: Infection prevention and control
OSCE: Objective Structured Clinical Exams
PAPR: Powered air purifying respirators
PPE: Personal protective equipment
